# The role of reflexivity in exploring exclusion in GP training: a qualitative study of GP educators

**DOI:** 10.3399/BJGPO.2025.0036

**Published:** 2026-02-11

**Authors:** Frances Wedgwood, Simon Bailey, Nagina Khan

**Affiliations:** 1 GP School, Kent Surrey and Sussex, NHS England South East (Workforce Training and Education), Reading, UK; 2 Centre for Health Services Studies, University of Kent, Canterbury, UK; 3 Centre of Health Services Studies, University of Kent, Canterbury, UK

**Keywords:** reflexivity, exclusion, GP training, postgraduate education, general practice, education, primary health care

## Abstract

**Background:**

Reflexivity is an under-researched concept in primary care education and educators receive no formal training in reflexivity. Evidence from other disciplines suggests that reflexivity can promote patient safety and inclusivity, making it a potentially valuable tool in medical training.

**Aim:**

To examine the use of reflexivity in GP training and how it can be used to explore experiences of exclusion.

**Design & setting:**

A qualitative analysis of GP educators’ perspectives in London and South East England.

**Method:**

Fourteen primary care educators were recruited. Focus groups and semi-structured interviews were conducted. Participants' opinions of reflexivity were explored, focusing on their own reflexivity, and that of their learners and the medical faculty. Data were transcribed verbatim and thematically analysed.

**Results:**

The following three key themes were identified: the value of reflexivity; revealing unfairness through reflexivity; and tokenistic reflection versus creative reflexivity.

**Conclusion:**

This study indicates GP educators are not familiar with the concept of reflexivity but are keen to consider how it could impact their learners. Moreover, the research demonstrated how issues of diversity, inclusion, and exclusion are brought to light through reflexive practice, and how this affects international medical graduate (IMG) learners. The participants identified a lack of organisational reflexivity as an important factor affecting inclusion and differential attainment, and it was suggested there was an ‘inverse education law’. The educators called for more diverse leadership, less tokenistic reflection, and more reflexive and creative learning tools to address the findings of this study.

## How this fits in

Exclusion in education can be defined as systemic practices that limit equitable access to learning opportunities and may manifest as unequal treatment, differential attainment in assessments, or curriculum bias. There is a lack of research in the field of postgraduate primary care education on educator understanding of reflexivity and how it might bring to light exclusion. This study was interested in investigating whether reflexivity and reflexive practices could help reveal instances of exclusion in GP training. Given the ongoing attainment gap that affects minoritised GPs in training, understanding how reflexivity both surfaces and challenges exclusion is critical to fostering more inclusive training environments. To achieve this aim, the GP educators in the study called for novel and creative approaches and embracing artificial intelligence (AI), as well as prioritising group work, identifying role models, and implementing considerable structural change to training.

## Introduction

Reflexivity has been defined as *‘recognising one’s own position in the world, both to better understand the limitations of one’s own knowing and to better appreciate the social realities of others’*.^1^ Although its origins as a term go back to the works of sociologists John Dewey and Robert Merton in the 1930s, reflexivity is still a concept that is relatively new to medical educators and poorly understood. In their review of reflexivity, Kenway and McLeod acknowledged the enormous influence of Pierre Bourdieu, a prolific French sociologist, who emphasised the role of reflexivity in understanding how individuals and groups navigate social structures, norms, and practices*.* ^2^ Bourdieu defined the concept of ‘habitus’, the set of learnt dispositions and practices that individuals acquire through socialisation and ‘field’, a social space or arena where individuals and groups interact and compete for various forms of capital, such as social status, economic resources, and symbolic recognition. Bourdieu argued that reflexivity involves becoming more aware of how the relationship between habitus and field influences one’s thoughts and actions and recognising objective constraints that shape individuals' lives, while also being aware of how these constraints are interpreted and acted on subjectively.

Within medicine, reflexivity has been considered until recently as something that only impacted research. However, recent research into how teams operate has argued that strengthening clinicians’ capacity for reflexivity in healthcare environments, addresses both clinicians’ psychological safety and inclusivity.^3,4^ Increasingly concepts or reflexivity are also starting to influence medical education theory.^5–7^ A recent scoping review identified a wide variety of educational strategies to enhance reflexivity in medical education.^8^ It showed significant evidence that educators encourage their learners to recognise their own experiences of both privilege and disadvantage, but reflective practice was not used in consistent ways across more than one study. Even studies that used a common approach, such as immersive experiences or reflective writing, used the approaches very differently, with little evidence to indicate which are the most effective strategies.

However, there is little research on reflexivity within primary care postgraduate education. Goldie (2017) demonstrates that our current GP training programmes do not foster reflexivity.^9^ A recent study of GP educators teaching on an undergraduate course on indigenous health highlighted a lack of knowledge about reflexivity and there was concern that this would negatively impact the students’ learning environment.^10^

In this paper, we examine how reflexivity might be used to explore exclusion in GP training, a topic not previously examined in the literature. In this context exclusion refers to systemic or interpersonal practices that limit equitable access to learning opportunities, often shaped by factors such as socioeconomic status, race, ethnicity, or disability.^11,12^ These forms of exclusion may manifest through unequal access to resources, biased interactions, or curricula that fail to reflect diverse experiences. Understanding how reflexivity can surface and challenge such dynamics is critical to fostering more inclusive training environments.

## Method

This paper draws on a qualitative study conducted as part of a master’s thesis that investigated the extent of primary care educators’ knowledge and understanding of reflexivity. Considering the lack of current knowledge about reflexivity in postgraduate GP education, an exploratory approach was adopted, combining narrative inquiry and constructivist grounded theory. Constructivist grounded theory is a qualitative research methodology that builds on traditional iterative grounded theory, while emphasising the importance of acknowledging the subjective perspectives and interpretations of both researchers and participants.^13^ Also used in qualitative research, narrative inquiry is a qualitative research methodology that explores the stories people tell to gain insights into their subjective experiences and the meanings they attach to those experiences.^14^ Given the subjective nature of reflexive thinking, these methodologies combined well.

A convenience sample of 30 primary care educators were invited from London and Southeastern England. In total 14 participants were recruited. All the participants were GPs, and there were eight females and six males, with educational experiences ranging from 3–30 years. Ten were UK graduates, four qualified overseas, eight were of minority ethnic background. An initial focus group was arranged to pilot the interview schedule, which drew on the work on reflexivity of Ng *et al* ^1^ and Fook and Askeland.^15^ The remaining participants participated in a further focus group and semi-structured interviews. The focus groups enabled points of comparison, consensus, and dissensus to be identified and discussed but it was recognised that it was harder to access individual experiences and perspectives and more intimate discussions; the semi-structured interviews allowed more controversial and sensitive topics to be aired.^16^

The focus groups were 90 minutes in length, and the interviews were 45 minutes. They were conducted over Microsoft Teams between June 2023 and August 2023 and used a schedule of open-ended questions, providing flexibility for participants to guide the focus of discussion. Whenever a new theme or idea emerged during the interviews, it was pursued. Data collection and data analysis occurred simultaneously as informed by Srivastava and Hopwood’s iterative framework, which supports the refinement of inquiry as understanding deepens.^17^ This approach enabled us to respond to emerging themes in real time by adapting the interview schedule; for example, adding questions that more deeply explored issues of exclusion. This iterative process enhanced the depth and relevance of the data collected.

We analysed the emerging narratives, through a process of inductive coding, identifying quotes and instances in the dataset that were similar in concept. At the end of the data collection period no new codes were introduced. These codes were organised into meaningful groups or sub-themes and subsequently grouped into broader themes. These themes are presented below.

The authors also needed to consider their own reflexivity: FW has been a primary care educator for many years, working with GP learners, many of whom are international medical graduates (IMGs). This involved maintaining a reflexive practice of personal writing throughout the study, continually questioning assumptions and motivations, which was shared in supervision meetings.^18,19^

## Results

The analysis uncovered the following three key themes: an emerging realisation of the value of reflexivity; revealing unfairness through reflexivity; and tokenistic reflection versus creative reflexivity. The themes and a selection of illustrative quotes are presented in Table 1.

**Table 1.** Themes and illustrative quotes

Theme 1: The value of reflexivity	
*1a*	*‘Having learned more about it I'm convinced that reflexivity is important for promoting inclusion. This is beyond reflection. This is about us consciously thinking about our values, our judgments and our perceptions and changing them.* [*…*] *That’s crucial in the context of the IMG workforce.’* (GP9-Interview)
*1b*	*‘I thought we were reflecting on why we hadn't recruited,* […] *but actually, I've been thinking about what it is about me that meant we haven't recruited to the roles that we wanted to recruit to. I think that was myself trying to work out what being critically reflexive is.’* (GP5-Focus group)
*1c*	*‘The more I read* [about reflexivity]*, the more I realised, it is about doing what most of us already do — become thinking doctors. We think about patients, … our interactions with colleagues.’* (GP12-Focus group)
*1d*	*‘I said to him* [...]*, “Well, the elephant in the room is you’re a white male, and she was expecting to see someone of colour.” Ten years ago there’s no way I would have had the confidence to say, “It’s because you're a white heterosexual man that this Black African woman did not get on with you” He just couldn't believe it.’* (GP10-Interview)
*1e*	*‘If anyone with alcohol or drug dependence comes in, I spend a lot more time with them.* […] *I feel they should have the time because they don't often get it. But when I thought about it, I realized that actually it’s because one of my relatives was an alcoholic.’* (GP7-Interview)
*1f*	*‘Although I came to this country as a teenager, I'm not an IMG. I'm a UK graduate so I can only have some experience of the journey* [the IMGs] *might be taken through, not fully.’* (GP9-Interview)
*1g*	*‘I didn't realise that the perception was that I'm quiet* [...] *I'm never short of words so I was quite surprised! Because in meetings — I thought — who wants to listen to the trainee? But it was cultural. I didn't realise — I was expected to speak and share.’* (GP11-Focus group)
*1h*	*‘I think* [reflexivity] *increases with awareness of self. How much we know, how much we don't know, and how much we're willing to grow to get to that point of finding value through reflection, I suppose.’* (GP4-Focus group)
*1i*	*‘Sometimes I can do it because I understand how my culture and my thought process can influence my consultations. Unless I have an appreciation for their culture and background, it might be difficult for me to signpost that trainee to challenges that they're having.’* (GP7-Interview)
*1j*	*‘If you’re feeling. “Oh, I can't reflect on this. I have to reflect on things that do not bring out my biases or vulnerabilities”, perhaps those things might not come out and they might be huge barriers to learning or someone’s professionalism in the future, if you've not been picked up quite early on.’* (GP4-Focus group)
*1k*	*‘It’s allowed me to, particularly when there’s a tough situation a trainee is in, to push a pause button and have a template, a go-to process that you refine over time and keep improving on.* [...] *And I always feel that that if it allows me, my trainees, my colleagues, to move forward and grow, then I think there’s immense value.’* (GP14-Focus group)
**Theme 2: Revealing unfairness through reflexivity**	
*2a*	*‘In places like India and Africa you need to be highly, highly academic to get into medical schools. So, these motivated trainees, when they move to the UK, because of the systemic and institutional issues they face, they're being challenged. There’s a perception that these trainees are somehow not good enough.’* (GP9-Interview)
*2b*	*‘I think it’s a really important part of our role as educators to recognise the power we hold, and the vulnerability that goes with that. People can feel very vulnerable in this unless we create very clearly a safe space.’* (GP12-Focus group)
*2c*	*‘We work on a deficit model. We're only looking for things that you're not getting right, rather than things you are getting right. It’s hard to admit to not doing things well when you're working in a structure where those things are going to be jumped on.’* (GP8-Interview)
*2d*	*‘Well, the exams* [...] *are racist. It must be quite demoralising to know, “I'm sat next to someone who’s got a 98% chance of passing first time. And I've got a 40% chance”.’* (GP10-Interview)
*2e*	*‘That’s the institutional bias, because as inclusive and understanding as I might want to be about my trainee’s way of practicing because of their background and influences, I'm still assessing them based on what the mark sheet says.’* (GP7-Interview)
*2f*	*‘We know that primary care is systemically racist, possibly more even than hospital.’* (GP8-Interview)
*2g*	*‘There were some in the room that were really shocked. “Well, it’s not that. You're calling me and my organization, and my country racist!” They weren't willing to engage in the thought that it could be something to do with discrimination rather than ability.’* (GP10-Interview)
*2h*	*‘So, you got a brown Prime Minister, a brown Home Secretary and lots of BME people in leadership who haven't improved the outcomes for the community that they've come from. That’s partly because of structural racism, because if you act in a different way to the organisation you're part of, you're side-lined. Even the organisations that are supposed to represent doctors have issues with leadership and structural racism.’* (GP9-Interview)
*2i*	*‘In* [named city] *now there are more GPs who are BME than are white. But whether that’s changed the power structures at all, it'd be interesting to know’.* (GP8-Interview)
*2j*	*‘I have trainees traveling miles in order to come to my training practice* [...] *just because they have lower scores I guess* [...] *They are just set up to fail, really.’* (GP3-Focus group)
*2k*	*‘It’s the inverse care law of education, isn't it?’* (GP10-Interview)
*2l*	*‘Their insights got less after* [Bawa-Garba]*. They stopped being self-aware. They stopped talking about their emotions.’* (GP13-Focus group)
**Theme 3: Tokenistic reflection versus creative reflexivity**	
*3a*	*‘There’s pressure in this “factory” to produce a “package” at the end of 3 years; the pressure on these wonderful young men and women, who have a lot of potential, can be quite erosive.’* (GP14-Focus group)
*3b*	*‘Get rid of recruitment scores and mix it all up. And by diversifying, it’s better for patients, better for the training program.’* (GP10-Interview)
*3c*	*‘I think IMGs have lots more anxiety because they feel that they're not doing well, just because they're not writing this wonderful prose for the trainers to look at.’* (GP3-Focus group)
*3d*	*‘A few years ago, I would have been very positive about reflective writing, but now it’s become a “something that you have to do in an e-portfolio” that people are going to mark. And actually, realistically people don't even read it.’* (GP8-Interview)
*3e*	*‘We moan and* [in panel] *someone might make a comment like “I think your entries could be more reflective,” but I've never heard of a trainee failing for not being reflective.’* (GP1-Focus group)
*3f*	*‘I think we'll move away from reflective writing because of ChatGPT, and start having “assessed reflective conversations,” which I don't like the idea of either. Your supervisor will say “We're going to have a reflective conversation and I'm going to assess you on it”.’* (GP8-Interview)
*3g*	*‘What you feel today, you might feel even tomorrow, but actually* [...] *in 5 or 10 years’ time, who wants that fairly inane thing you may have written down with great feeling, to still exist?’* (GP2-Focus group)
*3h*	*‘Maybe we should get much better at verbal reflection and reflexivity,* [...] *because if somebody else listens and really hears, that is terribly important in the moment.’* (GP3-Focus group)
*3i*	*‘Sometimes when you're talking out loud about problems, you arrive at the answer yourself without having needed anyone else to be involved in that conversation.’* (GP7-Interview)
*3j*	*‘Or an AI bot saying, “Did this consultation go this way because of you, and how you come across, what you stand for, your culture, your way of learning, your way of practicing medicine.?”* [...] *It starts the conversation.’* (GP10-Interview)
*3k*	*‘If you have an experienced facilitator who can include the views of the quietest member of the group. I feel that there’s empowerment implanted there.’* (GP14-Focus group)
*3l*	*‘Let’s mix these groups up … at least you've got the diverse conversations now. You don't have to be friends, but you listen to each other’s different opinions because you're not alike now.’* (GP13-Focus group)
*3m*	*‘When I was a trainee, I didn't see the value of small group work. But when I became GP, I was so glad to be part of those discussions, because that’s where you kind of unpack.* [...] *Even for like 20 minutes once a week where you kind of bring in some cases, to discuss, reflect on, in a very safe space.’* (GP9-Interview)

### The value of reflexivity

The first theme explores how the participants responded to the concept of reflexivity and how this evolved into their emerging realisation of its value in enriching the role of the primary care educator. Reflexivity was a relatively new concept to most of the GP educators in the study:

*‘I'll be honest, after you told me you want to talk about reflexivity, I went and read up about it* [...] *I would say this is a completely brand-new area for most of us.’* (GP9-Interview)

But they embraced the concept with ease, seeing how it affected their relationships with their learners but also within their day-to-day work consulting with patients. Several argued that reflexivity is what kept them and their patients safe and could prevent educator burnout. They also identified how reflexive practices could promote inclusion as both educators and clinicians see quotes 1a–c in Table 1).

The participants agreed a reflexive educator workforce was vitally important and wanted more training to deepen their understanding of reflexivity. Some expressed concern that their educator colleagues at all levels in the faculty did not understand the concept, given it is not currently addressed in the teaching courses for new educators in primary care. Some called for an extensive project to embed a more critical awareness of self and diversity, fearing that failing to do this would results in a loss of ‘*curiosit*y’, which could have considerable impacts on both the learning and clinical environment:

*‘If you can't get to some level of reflexivity as an educator, you're in danger of making all sorts of sort of mini judgments about people; you lose your curiosity about that and then I think you could get into trouble.’* (GP8-Interview)

In our discussions about reflexivity, participants tended to use personal or reflexive narratives to understand their own social locators, examining their own and interpersonal dynamics within social and cultural settings through the lens of retrospective autobiographical stories (see quotes 1d–g in Table 1). The participants described how this reflexive understanding of their own narrative, and how it would ‘*talk*’ to the learner’s narratives, might be explored in the educational supervision, or tutorial, a fertile ground for exploring and re-making narratives. By bringing forth new stories the educators are exploring new territories of critical understanding. Failing to explore these narratives was dangerous:

*‘If you don't know what your own narrative is, and particularly your own meta-narratives about your social and cultural upbringing and how that affects how you think and feel, I think it’s very difficult to put yourself in the position of people who have different meta-narratives and see how those might talk to each other.’* (GP8-Interview)

Several participants mentioned the peer support groups that GP educators participate in as a site of productive discussion, and it is likely that this resource had given many of these educators an informal grounding in reflexivity. Usually known as trainers’ groups and these provide a supportive environment where members can share their experiences, dilemmas, and emotional reactions related to educational issues. These groups help educators gain insight into the complex dynamics of educator–learner relationships, and narrative-making is often a part of this process:

*‘It helps triangulate your opinion of the trainee; how they perceive the world around them, how the world sees them … which can help when you're making judgments on professionalism, on their engagement, or their enthusiasm for learning.’* (GP4-Focus group*)*

### Revealing unfairness through reflexivity

The second theme focused on how exploring reflexivity brought forth narratives of inequity and exclusion. A recurring anxiety among the study participants concerned their learners’ vulnerability and risk, and the suspicion that reflexivity is lacking in our institutions, leading to more harsh treatment for doctors from minority ethnic groups and IMGs in particular (see quotes 2a–c in Table 1). The study participants all shared a strong sense of distress in response to issues of inequity and exclusion. This was particularly noticeable in the interviews, in which all the participants explicitly mentioned racism. Several felt acknowledging the role of reflexivity in institutions meant acknowledging systemic racism (see quotes 2d–h in Table 1). They felt the role of primary care educators in promoting culturally diverse and inclusive medical education was crucial, but they felt anxious and under-prepared by current diversity programmes, as well as concern that responding to their learners’ difficulties was leading to educator burnout. The participants also frequently mentioned the concern about the need for role models and mentors, and the lack of diversity in leadership positions (see quote 2i in Table 1).

In the discussions around safety, some participants felt that unless educators encouraged students and trainees to embrace vulnerability, they wouldn’t reach that point of knowing about themselves. But this level of vulnerability required for more inclusive practices left many of the educators very anxious, particularly concerning the power dynamics involved and they emphasised how reflexivity in their learners could not happen without safety and support:

*‘I don't think true reflexivity can possibly happen unless you are in a very, very safe environment, and what you're writing down, you know, to be “safe”, because it’s actually deep and personal and honest.’* (GP2-Focus group)

The participants acknowledged their difficulties discussing racism and issues of white privilege. But they perceived the biggest barrier to inclusion was a lack of organisational reflexivity leading to fewer opportunities for trainees who did not fit the mould, and IMGs in particular. The participants noted that disadvantaged groups frequently have increased levels of responsibility, caring for children or other family members, and that the programme made little concession towards trainees’ individual situations.

*‘If your experience of institutions is they're not gonna support you, because of your neurodiversity, ethnicity, gender, sexuality, language, then I think you're gonna find it harder.* [...] *And so I think we are failing those trainees if I'm honest.’* (GP10-Interview)

Several participants went as far as to call this institutional bias, and listed several examples, such as preferencing the trainees’ recruitment scores over all other considerations when placing them was particularly unfair on IMGs (see quote 2j in Table 1), who either needed to move or travel great distances, unlike their UK counterparts. One participant invoked the inverse care law (see quote 2k in Table 1), suggesting the same is true in medical education; those with less support needs have better training environments, while those who are most in need of support end up training in the most challenging, deprived areas. The memory of what happened to the trainee paediatrician Hadiza Bawa-Garba, also an IMG, whose portfolio entry was used against her in court,^20^ was recalled repeatedly (see quote 2l in Table 1). The underlying fear was that educators were asking learners to be open and vulnerable, but structures lacked the reflexivity to support doctors if things went wrong:

‘*The GMC is not known for its kindness when you admit to a mistake, even if you can understand why it happens. So, on one level we're saying, let’s open up to this, let’s learn from it, but ultimately if you do admit something, sometimes you can be in deep shit. Really.’* (GP8-Interview)

### Tokenistic reflection versus creative reflexivity

The third theme identifies a frustration with current reflective practice and a shared drive to devise creative and meaningful solutions adapting reflexivity so it could foster inclusion and diversity in primary care training. Several called for significant change such as free exams, or no exams, more diversified training schemes by abandoning the recruitment scores, less time in hospital, and longer training (see quotes 3b in Table 1). In addition to their thoughts on structural change, they also wanted educational tools that were creative and inclusive, unlike mandated reflective writing, which they felt had become tokenistic, a ‘*tick-box exercise*’ that disadvantaged and excluded vulnerable learners (see quotes 3c–e in Table 1). They had several interesting ideas about how this could be done. Verbal discussions often felt safer, particularly considering the permanence of the written reflection. Furthermore, these reflexive conversations had the potential to encourage self-examination of learners’ privilege and oppression (see quotes 3g,h in Table 1). More mentoring was also mentioned as an option and identifying role models for IMGs. Many of the participants had used novel ways to capture a moment of reflexivity, such as *‘walking the neighbourhood*’ instead of a conventional tutorial, or using a voice note rather than the confining structures of assessed written reflections. Several had used artistic expressions to help learners think reflexively about their medical journeys. They discussed how they used multiple different artforms, such as galleries, plays, book clubs, or art-making:

*‘Maybe they can speak it. Or write a play or a poem about it. Or draw it. Draw what you did. What could you have done differently next time?’* (GP13-Focus group)

A common topic that was raised was how artificial intelligence (AI) might be used to promote reflexivity. Although concerned that learners might use AI to *‘cheat*’ when writing in their learning portfolios, educators were also interested how it could be used for learning. Several had already experimented with AI, using it as an instructive tool to show trainees what reflexive writing could look like. One participant suggested how AI tools could guide trainees through the steps to develop their own reflexive thinking (see quote 3j in Table 1). Another recalled helping an IMG rewrite a more empathic response to a complaint, considering the patient’s viewpoint:

*‘So what I'm trying to do with them is, you write your entry first and then put that entry in ChatGPT and see what it says and improve.’* (GP9-Interview)

The participants agreed universally that more group work exploring ideas collectively and discovering how others view the world, would promote more reflexive thinking and inclusion (see quote 3k,l in Table 1). They reflected on their own experiences of reflexive group learning and how it can help move thinking forward and reflect the inequities and power dynamics of society, although they joked that the trainees often see group work as less valuable in comparison with lectures (see quotes 3m in Table 1). In addition, they recognised that the pressures to pass the Royal College of General Practitioners (RCGP) examinations would often blind the learners to the opportunities of group working. However, they acknowledged that even group work could undermine inclusion if the facilitation was poor and the educators biased, and to include everyone this had to be challenged:

*‘Are we challenging our fellow educators when we're hearing or seeing things that are not acceptable? Inclusion is about creating that belonging. But the people running the groups have to foster that! It’s not OK!’* (GP12-Focus group)

The research findings and suggested solutions for the issues raised are summarised in Table 2. The possibilities and drawbacks of these approaches is suggested in Table 3.

**Table 2.** Summary of findings and recommendations

Findings	Recommendations
GP educators are generally unfamiliar with the concept of reflexivity but are aware how their culture and background affects the judgements they make They are keen to know more and expressed concern about the need for a more reflexive and less prejudiced workforce GP educators are comfortable with reflexive narratives, instinctively linking their own experiences to their work as clinicians and educators They recognised the value of reflexivity in educator–learner relationships, using it to tease out blind spots and sharing their own limitations to help disentangle challenging moments	Embed teaching on reflexivity into current training programmes for educational supervisors Consider teaching about reflexivity in conjunction with current diversity teaching Continue to support and fund peer-mentoring groups such as trainers’ groups Prioritise the weekly tutorial, identified in the research as one of the most fertile areas for promoting reflexivity
GP educators were very concerned about the vulnerability of their learners in their written reflections, particularly following Bawa-Garba They call for more reassurance around psychological safety for learners They identified that structures often unfairly impacted on IMGs Several educators identified a lack of organisational reflexivity and identified structural racism withing GP training structures	Educational and clinical supervisors to recognise the vulnerability of their learners Easier access to existing wellbeing services Consider more targeted support for IMGs around induction, linguistics, and seeking help that continues throughout the training RCGP and DHSC to consider how to encourage more diverse leadership within primary care
GP educators have concerns about mandated reflective writing, and believe it adversely impacts IMGs who do not have the linguistic capital AI could upskill IMGs’ writing, but there is concern that the reflections would no longer be genuine or reflexive GP educators often prefer reflective conversations that feel safer or private reflections that are not shared They suggested creative and innovative alternatives, for example, voice notes, AI, and other creative projects to encourage reflexive thinking Although learners often prefer lectures, GP educators felt that group work was superior and could develop more reflexive thinking GP educators would like to see structural change such as free exams, or no exams, more diversified training schemes by abandoning the recruitment scores, less time in hospital and longer training	RCGP to consider the value of mandated written reflection in the era of ChatGPT Continue to support the value of tutorial discussions and the case-based discussion in promoting reflexivity Medical faculties to recognise the value of innovative and creative educational tools and promote these in TPD conferences and educator workshops Prioritise group work with good facilitation in the half-day release Consideration of longer training for GPs and abandon the use of assessment scores in recruitment and allocation of GP trainees

AI = artificial intelligence. DHSC = Department of Health and Social Care . IMG = international medical graduate. RCGP = Royal College of General Practitioners. TPD = Training Programme Director

**Table 3.** Suggested solutions for fostering reflexivity in GP training

Suggested solutions to foster reflexivity	Opportunities	Drawbacks
Encouraging reflexive conversations	Tutorials and role play Adapting the case-based discussion to encourage critical self-examination	Learners may be vulnerable and exposed if educators not reflexive in response Concerns around privacy and security, for example, notes of conversation
Embedding reflexivity into new educator training	Introduce modules on reflexivity into new educator courses	May result in superficial reflexivity
Developing reflexivity in existing educators	Using trainer groups Workshops in training programme director and educator conferences	Educator cynicism and burnout
Encouraging more group work	Established technique Can be integrated into existing VTS sessions	Needs expert facilitation to encourage reflexive thinking Needs protected time Not valued by learners
Identifying mentors	Highly valued by vulnerable learners Informal mentoring already takes place	Costs involved identifying, recruiting, training, and renumerating mentors Ensuring everyone has access
Creative or artistic projects	Collaborating with artists, actors, musicians, and so on	Scepticism from learners Educators maybe reluctant to lead sessions
Using AI	Using AI to improve reflective writing Using AI to pose questions to explore critical self-examination	Concerns regarding inauthenticity Concerns regarding privacy and data security
Voice notes	Quick, easy, authentic Can be uploaded into the eportfolio	Needs transcription (but AI should help with this task)

AI = artificial intelligence. CBD = case-based discussion. VTS = Vocational Training Scheme. TPD = Training Programme Director

## Discussion

### Summary

In this paper we set out to examine the use of reflexivity in GP training and how it can be used to explore exclusion. Although they did not recognise the term, the educators already demonstrated their ‘*continual questioning of the assumptions underlying one’s ways of being, seeing, and thinking’,* as described by Ng *et al*.^1^ We observed the educators working through the reflexive process, using reflexive narratives.^21–23^ Their prior experience of educational supervision, and the Balint model of peer support, processes that encourages reflexive thinking in both educator and learner, helped them gain insight into the complex dynamics of educator–learner relationships.^24,25^

The thematic analysis of their responses uncovered multiple issues of diversity, inclusion, and exclusion that are brought to light through reflexive practice. Furthermore our research suggests that there is a shared drive among GP educators to foster and cultivate reflexivity; suggestions for doing this include prioritising group work and reflexive conversations in tutorials, identifying role models and mentors, and devising creative and innovative reflexive learning tools rather than tokenistic reflection. This could positively impact the experiences of vulnerable and minoritised learners, in particular IMGs, who make up more than 50% of current postgraduate specialist doctors training to become GPs in the UK, and often have little NHS experience before starting training.^26^ This group of doctors were highlighted repeatedly as more vulnerable in our research and recent Ipsos data confirms IMGs in GP training are significantly impacted by a combination of significant challenges, relating to induction, relocation, navigating the visa system, unfamiliar and complex training demands, and a reluctance to seek help.^27^ Furthermore, this group is overwhelmingly affected by differential attainment, the gap being particularly pronounced in general practice; however, it is still not clear which of the many interventions trialled are the ones most appropriate to eliminate the gap.^28–34^ Our research suggests that fostering reflexivity could transform the learning environment but also could contribute to the plan for tackling differential attainment. Future research could involve piloting some of the suggested interventions within GP training schemes and evaluating outcomes for learners.

### Strengths and limitations

These preliminary findings provide a unique insight into postgraduate educators’ understanding of reflexivity and how it impacts inclusion. The research generated a richness of narrative material, but is limited by the small study size, and participants who opted in to the study, which may have introduced selection bias as they may have had a pre-existing interest in the topic. As this research was undertaken for a master’s dissertation, time limitations also impacted the sampling of the data. It would be useful to widen this study across the UK, gathering data from a variety of educators of different disciplines, and IMGs and other learners at various levels of training.

### Comparison with existing literature

We could find no published research in the field of postgraduate primary care education concerning educator understanding of reflexivity and how it might reveal exclusion. However, many studies in the current literature analyse attempts to introduce reflexivity into undergraduate medical education. At Stanford University, complementary programmes of ‘culturally reflexive medicine’ have been introduced for minoritised undergraduates using journaling, mentoring, and peer groups to address the unique tensions they experience.^35^ Elsewhere innovative methods are employed to promote reflexivity, such as taking students on tours of disadvantaged neighbourhoods followed by reflexive writing exercises,^36^ or using reflexive educational tools to deepen understanding of curricular areas where there is disadvantage; planetary health,^37^ social accountability,^38^ decoloniality,^39^ or indigenous health.^40^ The need to support novice educators, who lack confidence in reflexive methods and often prefer didactic teaching styles, has also been identified.^41^ These studies further support our argument that there is a need to develop reflexivity within postgraduate primary care education.

Rather than viewing reflective practice as a pedagogical tool that results in learners becoming ‘*reflective zombie*[s]*’,* it has been suggested educators use it as a focus for critical inquiry, abandon the ‘*checklist approach’* and instead embrace the diversity and personal aspects of the reflexive process.^42,43^ This could involve drawing on the humanistic talents of the arts and humanities as our participants suggested. Koch (2018) argues that medical and natural sciences do not nurture the reflective methodologies that permit their scrutiny and assessment, whereas the humanities subject the reflexive aspects of medicine to interrogation and discussion.^44^

Suggested creative methods for developing reflexivity such as using art-making or music has a good evidence basis; research has found art-making assists undergraduates in examining their vulnerability and professional identity, much like the critical engagement that reflexivity calls for.^45,46^ There is currently less evidence around using AI in medical education but the research is developing rapidly,^47^ and there are already examples of how ChatGPT can be used as a digital Balint to develop reflective practice in GP training .^48^ Vlogging, similar to voice notes, has already been shown to deepen critical reflection among undergraduates.^49^ However, a more rigorous analysis of the risks and benefits needs to be undertaken, to ensure the safety and privacy of learners.

The lack of role models and diverse leadership was also highlighted in our research; this has been identified as an urgent problem at undergraduate level that impacts attrition, performance, and future career choices.^50,51^ Our participants also called for more prioritisation of group work within training to help develop reflexive thinking, which aligns with evidence that collectively exploring ideas and discovering how others view the world through group work is better at developing critical thinking than didactic teaching methods.^52,53^ Our participants specifically called for better facilitation; this is essential to model constructive criticism and respectful disagreement and co-create common ground rules that mitigate the social inequities and power dynamics that are invariably imported into the group.^54,55^

Our research suggests developing reflexivity in our workforce could be a more productive way to promote inclusion than current diversity programmes, which when handled poorly and insensitively, can be traumatic, something that has been referred to as *‘educational iatrogenesis’*.^56–58^ Recently, there is concern that unless educators get it right, formal teaching about oppression risks replicating harm and propagating othering and the culture of bias will remain.^59^ Our research also raised the possibility of an ‘inverse care law’ of medical education, whereby those with less support needs have better training environments, while those who are most in need of support end up training in the most challenging, deprived areas. This has been observed elsewhere, but more research is required to explore this concept.^60,61^

### Implications for practice

This research demonstrated how exclusive practices are brought to light through reflexive practice, and how this affects IMGs. It indicates the need to foster both educator and organisational reflexivity to understand and respond to learners’ diverse educational needs; given the ongoing attainment gap, this is an important and novel area of study. To address these challenges, introducing reflexive activities to all stages of GP education using creative and meaningful solutions should be given urgent consideration.

## Data

The dataset relied on in this article is available from the corresponding author on reasonable request.
